# Development of an optimized sample preparation method for quantification of free fatty acids in food using liquid chromatography-mass spectrometry

**DOI:** 10.1038/s41598-021-85288-1

**Published:** 2021-03-15

**Authors:** Hyejin Park, Woo-Young Song, Hyeonjeon Cha, Tae-Young Kim

**Affiliations:** School of Earth Sciences and Environmental Engineering, Gwangju Institute of Science and Technology, Gwangju, 61005 Republic of Korea

**Keywords:** Mass spectrometry, Lipidomics

## Abstract

Accurate and precise determination of free fatty acid (FFA) contents is essential for quality control and assurance in food production. Herein, a mass spectrometric study was performed to develop a sample preparation protocol that can minimize exogenous FFA contamination during the quantification of FFA in food. The quantities of exogenous FFAs were measured using various combinations of seven pretreatment methods for a sample tube, three extraction methods, and four types of sample tubes. Methanol washing could effectively reduce exogenous palmitic acid (PA) and stearic acid (SA) by 73 and 64%, respectively, in contrast to furnace baking resulting in a decrease in the amount of PA and SA contaminants by 50 and 37%, respectively. A lower amount of FFA contaminants was extracted from glass tubes during comparative analysis of the four different sample tubes studied. A combination of a methanol-washed glass tube and chloroform extraction solvent was determined to be the optimal method for minimizing the error in FFA quantification. The optimized sample preparation protocol used for FFA quantification can lower the amount of foreign palmitic acid and stearic acid to the sub-nanomolar level in the analysis of FFAs in skimmed milk.

## Introduction

Fatty acid (FFA) present in food has diverse effects on health and nutrition as well as sensory characteristics of food, such as flavor and taste^1^. Thus, the accurate quantification of FFAs in food is critical for quality control and assurance in the food industry^2–5^. However, it is challenging to accurately and reliably determine the FFA contents in food because FFAs typically exist at low concentrations in the presence of highly complex matrices consisting of other nutrients, such as fats, proteins and carbohydrates^6,7^.

Various analytical techniques have been employed for the quantification of FFAs in food. The most commonly used method to quantify FFAs involves the chemical derivatization of FFAs into fatty acid methyl esters (FAMEs)^8^, followed by gas chromatography (GC) combined with either flame ionization detector (FID) or mass spectrometry (MS)^9^. Very recently, Mannion et al. proposed the conversion of FFAs into fatty acid butyl esters as a novel esterification strategy for the accurate quantification of FFAs in dairy foods^10^. Supercritical fluid chromatography^11,12^ and high-performance liquid chromatography (HPLC)^13–17^ have also been widely exploited to quantify either derivatized or non-derivatized FFAs.

Regardless of the analytical method used for quantification, the extraction of FFAs from food samples is a prerequisite step to reduce any interference from the food matrix in the downstream analysis steps. Folch^18^ and Bligh/Dyer (B/D)^19^ liquid–liquid extraction (LLE) methods, employing a mixed solvent consisting of chloroform, methanol, and water at various ratios, have been used as the standard procedure to extract FFAs. Matyash and coworkers suggested methyl-*tert*-butyl ether (MTBE) as an alternative organic solvent, which can occupy the top layer in two-phase LLE and facilitate the recovery of FFAs, while avoiding contamination with the aqueous phase^20^. LLE is an easy and efficient method used for the extraction of FFAs, but it may also be used to extract exogenous FFAs used as additives to improve the surface properties of plastic consumables^21^, such as pipette tips, sample tubes^22^, and barrels of solid-phase extraction columns^23^. Exogenous FFA contaminants are also found in soft polyethylene frits containing a plasticizer^24^, chromatographic papers used for blood collection^25^, glass products and even high-purity organic solvents^26^. Unfortunately, the amount of foreign FFA contaminants introduced during the sample preparation procedure is high enough to be detected in MS used for the quantification of FFA and complete elimination of these contaminants remains a significant challenge. The presence of FFA contaminants eventually leads to an overestimation of the endogenous FFA content, resulting in inaccuracies during the quantitative measurement of FFAs. Thus, it is of great importance to determine the amount of FFA contaminants originating from experimental reagents or apparatus and to set up an experimental procedure that can reduce the impact of exogenous FFAs on the quantification of endogenous FFAs present in food samples.

Herein, we have conducted a systematic study to develop an optimized experimental protocol that minimizes the amount of foreign FFA contaminants negatively affecting the accurate quantitative analysis of FFAs. The amounts of exogenous FFAs detected in LC–MS were compared with regard to the following three experimental parameters: (1) Pretreatment of the sample container, (2) extraction method, and (3) type of sample container. Finally, the optimal method established in this comparative study was exploited for the quantification of FFAs found in commercial milk samples using LC–MS.

## Materials and results

### Materials

HPLC-grade water, acetonitrile (ACN), and chloroform were from Thermo Fisher Scientific (Waltham, MA, USA). Palmitic acid (PA), stearic acid (SA), HPLC grade MTBE, *n*-hexane, ammonium hydroxide and formic acid were from Sigma-Aldrich (St. Louis, MO, USA). HPLC-grade methanol (MeOH) and ethanol (EtOH) was from DUKSAN (Ansan, South Korea). Isotope-labeled [methyl-d3]-PA (d3-PA) and [methyl-d3]-SA (d3-SA) were from Cambridge Isotope Laboratories Inc. (Andover, MA, USA). Plastic tubes were from Eppendorf (Ep) (Hamburg, Germany) and SPL Life Sciences (SPL) (Pocheon, South Korea), and glass tubes were from Glassco Laboratory Equipments (GLASSCO) (Haryana, India) and Supelco Analytical Products (SUPELCO) (Bellefonte, PA, USA).

### Evaluation of pretreatment methods for exogenous FFA analysis

Different pretreatment methods of sample containers were evaluated in terms of their efficiency toward eliminating background FFA contaminants in an empty tube. The first method was to bake a glass tube overnight at 450 °C in an electric furnace^26^. In the second method, a glass tube was rinsed three times under ultrasonication for 10 min in different organic solvents^22^. MeOH, EtOH, hexane, and a mixture of EtOH and hexane, which are commonly used for solvation of fatty acids^27^, were tested. The mixture of EtOH and hexane was chosen at the azeotropic concentration (18:82, w/w) because it possesses a constant composition during evaporation. In addition, to investigate the effect of pH on solvation of exogenous FFAs, 0.1% (v/v) of formic acid or ammonium hydroxide were added to EtOH as a pH modifier. These experiments were conducted with GLASSCO glass tubes that are durable at very high temperature. After the pretreatment steps, the residual FFAs were extracted using the Folch method, as shown in Table 1. The recovered organic phase was dried in a vacuum evaporator (Labconco, Kansas City, MO, USA) and resuspended in 100 μL of MeOH prior to LC–MS analysis. To evaluate the variability of exogenous FFAs in one package and between packages, three packages with different lot numbers and six tubes per each package were tested. All the other experiments were run in six replicates with tubes of the same lot number unless indicated otherwise. The detailed experimental conditions are listed in Table S1.

**Table 1:** Composition and ratio of the solvents used in the three different FFA extraction methods studied.

Folch	B/D	Matyash
+ 1.25 mL chloroform: MeOH 2:1 (v/v)	+ 0.75 mL chloroform: MeOH 1:2 (v/v)	+ 0.3 mL MeOH
	+ 0.25 mL chloroform	+ 1 mL MTBE
+ 0.25 mL water	+ 0.5 mL water	+ 0.2 mL water

### Comparison of the extraction methods and type of sample tube

To compare the level of exogenous FFAs extracted from commercially available consumables, two plastic tubes (Eppendorf and SPL) and two glass tubes (GLASSCO and SUPELCO) were subjected to three different FFA extraction methods (Folch, B/D, and Matyash) with some modifications. The composition and ratio of the solvents used for each extraction method are listed in Table 1. In brief, the corresponding amount of organic solvent (Table 1) was added to an empty tube and the tube was vortexed for 20 min. Subsequently, the aqueous solvent was added to the tube and the mixed solution was centrifuged at 500 g for 10 min to support phase separation. The detailed experimental conditions are given in Table S2.

### FFA extraction from skimmed milk

Skimmed milk samples were purchased from a local market in Gwangju, South Korea and stored at 4 °C prior to analysis. Two different conditions were tested for the extraction of FFAs from skimmed milk. The first was a general FFA extraction method without any pretreatment of the sample tube and the second was the optimized FFA extraction scheme that minimizes the level of exogenous FFA contaminants, which was established from the comparative experiments described in the above sections. The procedure used for FFA extraction from skimmed milk was carried out according to the method described by Scano et al.^4^ with some modifications. Briefly, 15 mL of the milk sample was sonicated for 15 min to disrupt the micelle structure of milk and 100 μL of the sample was then transferred to an Ep tube or MeOH washed GLASSCO tube. The FFAs were finally extracted using the Folch and B/D methods for triplicate samples.

### LC–MS analysis

FFAs were analyzed on an Agilent 6520 quadrupole time-of-flight mass spectrometer coupled with an Agilent 1260 infinity HPLC system (Santa Clara, CA, USA) after injecting 20 μL of a sample. Chromatographic separation was performed on a C8 column (XBridge BEH, 4.6 × 100 mm, 3.5 μm, Waters, Milford, MA, USA) using isocratic elution with a mobile phase consisting of 0.1% formic acid in water and 0.1% formic acid in ACN (13:87, v/v) for 5 min at a flow rate of 1 mL/min. The C8 column was used to eliminate the possibility of the carryover effect of long-chain FFA, instead of a C18 column, which is commonly used in lipidomics. The temperatures of the column and auto-sampler were maintained at room temperature and 4 °C, respectively. The ESI parameters were as follows: Sheath gas, 350 °C at 12 L/min; nebulizer, 60 psi; capillary voltage, 3.5 kV; fragmentor, 200 V. MS was performed in negative ion mode from 200 to 300 m/z with an acquisition rate of 2 spectra/sec.

### Quantification of FFAs

The FFAs were identified based on the accurate mass and retention time of their corresponding standards. For the accurate quantification of PA and SA, which are known as the two major FFA contaminants originating from the sample tube, d3-PA and d3-SA were spiked into the extraction solution as internal standards (ISTDs) at a concentration of 5 μM and the absolute concentrations of PA and SA were obtained using the ratio of peak area observed for STD:ISTD. Quantification of FFAs was performed using Agilent MassHunter Qualitative Analysis software (ver. B.05.00). The ratio of endogenous to exogenous FFA contaminants in the skimmed milk samples was calculated using the following equation: [Amount of exogenous FFA contaminant/ total extracted FFA (exogenous FFA contaminants + endogenous FFA)] × 100.

### Statistical analysis

To compare the FFA removal efficiency of different pretreatment methods, significant differences in the amounts of exogenous FFAs were tested by an analysis of variance (ANOVA). The data normality and homogeneity of variance were checked with Shapiro–Wilk test and Levene’s test, respectively. Since the amount of FFA was not normally distributed and its variance was not homogeneous, Kruskal–Wallis nonparametric one-way ANOVA was conducted. If a difference in the amount of FFAs among the conditions was found to be significant, Dunn’s post hoc analysis was conducted to reveal the pairs of significantly different conditions. For comparison of exogenous FFAs with and without pretreatment using different extraction methods and types of sample tubes, Welch’s t test for unequal variance was used. All statistical tests were conducted using R statistical software (Version 4.0.3, The R Foundation).

## Results and discussion

### Comparison of pretreatment methods used for the sample tube

Multiple pretreatment methods were tested to estimate how much of FFA contaminants in a sample tube can be reduced. First, GLASSCO glass tubes were baked in an electrical furnace overnight at 450 °C, which is higher than the boiling points of the FFAs analyzed, in order to evaporate the physically adsorbed residual FFA contaminants on the glass surface. Second, the glass tubes were sonicated for 10 min in various solvents three times to leach out hydrophobic FFAs.

**Figure 1:**
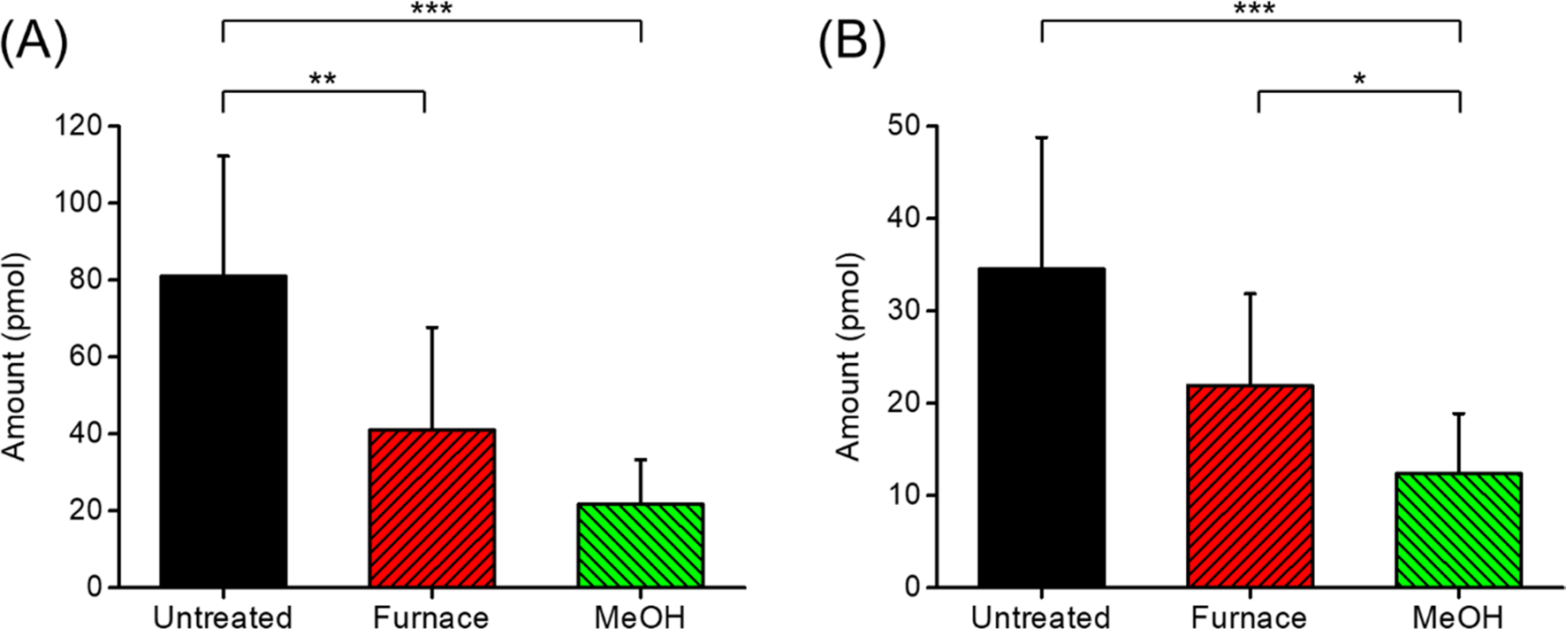
A comparison of the amount of exogenous (**A**) PA and (**B**) SA extracted from GLASSCO glass tubes with different pretreatment methods (furnace baking and MeOH washing). Data are shown as the mean ± standard deviation (*n* = 18). Asterisks denote statistically significant differences by Dunn’s post hoc test (*P < 0.05, **P < 0.01 and ***P < 0.001, respectively).

The amounts of PA and SA remaining in the tubes (number of replicates, *n* = 18) after application of furnace baking and MeOH washing are shown in Fig. 1. The PA contaminant amount was measured to be 81.0 ± 31.3 pmol without pretreatment and was reduced to 40.9 ± 26.8 and 21.7 ± 11.6 pmol upon using furnace baking and MeOH washing pretreatments, respectively (Fig. 1A). Both furnace baking and MeOH washing showed significant reduction of exogenous PA, but MeOH washing showed a greater removal efficiency than furnace baking. For exogenous SA, the concentration was 34.5 ± 14.3 pmol without pretreatment and was reduced to 21.9 ± 10.0 and 12.4 ± 6.5 pmol upon using furnace baking and MeOH washing pretreatments, respectively (Fig. 1B). MeOH washing significantly reduced exogenous SA, however, furnace baking had no significance effect on SA removal (*P* = 1.39 × 10^−1^ for furnace baking and 4.53 × 10^−6^ for MeOH washing, Kruskal Wallis test, Dunn’s post hoc test with Bonferroni correction). The poor removal efficiency of SA upon furnace baking is in agreement with the lower vapor pressure of SA compared to PA at equal temperature^28^. In contrast, a significant reduction of PA or SA was observed upon MeOH washing, resulting in an average of 73% and 64% removal for PA and SA, respectively. Figure S1 shows the inter-lot variation of exogenous FFA in untreated, furnace baked and MeOH washed tubes from three different packages (X, Y and Z) (*n* = 6 for each lot, *n* = 18 for total). The variation in the concentration of exogenous FFA among different packages was minor compared to that among individual tubes from a same package (*P* > 0.05, Kruskal–Wallis test). Thus, the rest of analysis was performed in a single batch, rather than three batches.

**Figure 2:**
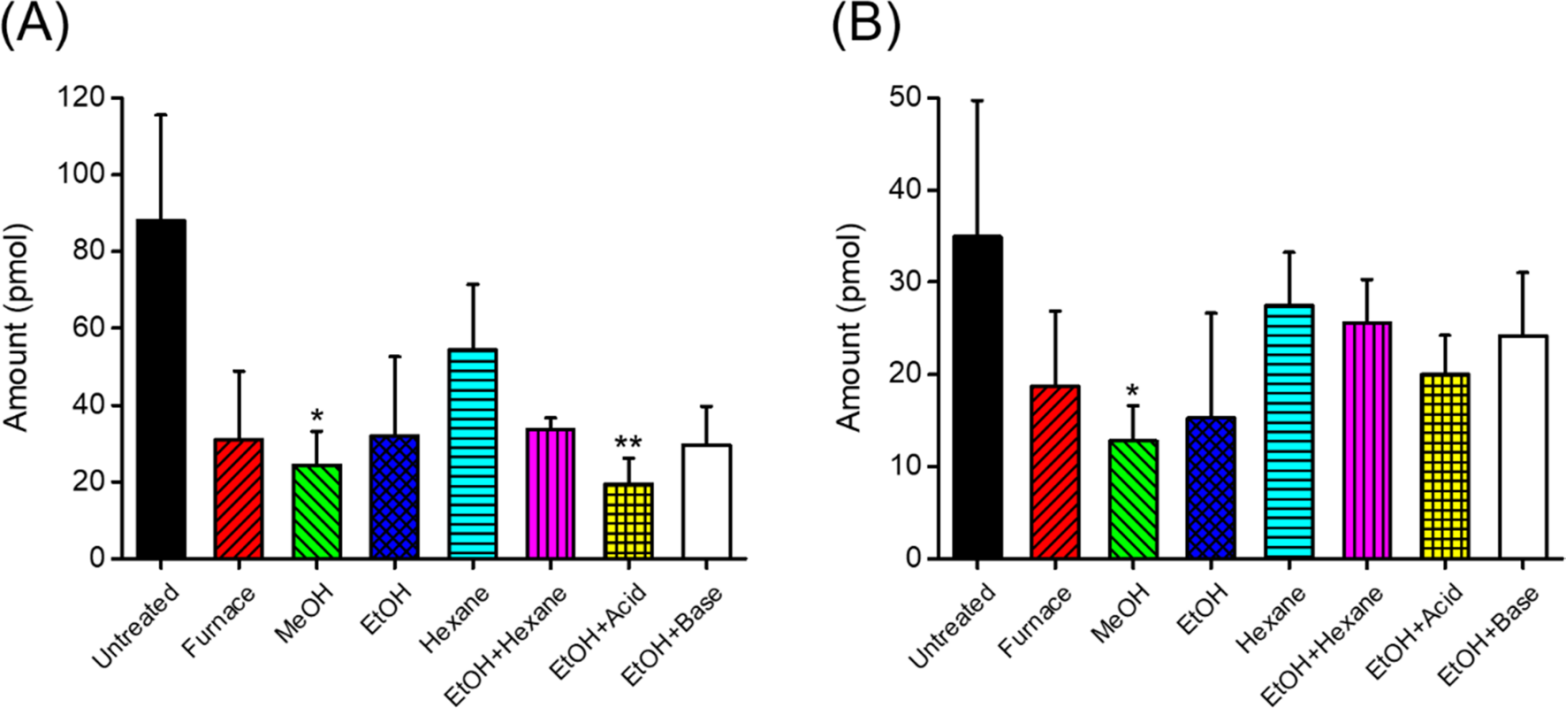
A comparison of the amount of exogenous (**A**) PA and (**B**) SA extracted from GLASSCO glass tubes with various pretreatment methods using different solvents. Data are shown as the mean ± standard deviation (SD) (*n* = 6). Asterisks denote statistically significant differences compared with untreated by the Dunn’s post hoc test (*P < 0.05, **P < 0.01, and ***P < 0.001, respectively).

The comparison between different pretreatment solvent types for exogenous FFA removal in sample tube (*n* = 6) is displayed in Fig. 2. MeOH washing significantly reduced exogenous PA and SA concentration (*P* = 2.72 × 10^−2^, Kruskal Wallis test, Dunn’s post hoc test with Bonferroni correction). Pretreatment with *n*-hexane was not effective for exogenous FFA removal, in spite of its higher solubility for FFAs over MeOH and EtOH^29^. Although the acidified EtOH could significantly reduce PA, it was not effective for SA removal. Since only MeOH washing showed significant reductions of exogenous PA and SA, it was chosen as the pretreatment method for further experiments to minimize the amount of exogenous FFAs extracted from the sample tube.

### Comparison of the different FFA extraction methods and sample tubes

To study the effect of the pretreatment used for the sample tubes on the conventional FFA analysis protocols studied, the MeOH washing method was applied to all combinations of the four sample tubes (Eppendorf and SPL plastic tubes, and GLASSCO and SUPELCO glass tubes) and three conventional FFA extraction methods (Folch, B/D, and Matyash) studied, and compared with the non-pretreatment method in terms of the FFA contaminants present in the sample tubes. The amounts of PA and SA extracted from empty tubes with and without MeOH washing are displayed in Fig. 2. SPL tubes showed the highest level of PA and SA contamination across the different extraction methods among the sample tubes studied. In contrast, Ep tubes had more than 10 times less PA and SA contaminants than SPL tubes although they are both plastic tubes. SUPELCO and GLASSCO tubes had even less amount of FFA in a range from 30 to 100 pmol. Using the B/D method, MeOH washing of GLASSCO tubes decreased the amount of exogenous PA and SA by 62 and 41% (*P* < 0.05, Welch’s *t*-test), respectively. For the Folch method, the MeOH washing step of GLASSCO tubes decreased the amounts of PA and SA contaminants by 37% (*P* < 0.05, Welch’s *t*-test) and 12%, respectively. Among the four different types of sample tubes studied, GLASSCO tubes exhibited the minimum absolute amount of residual PA and SA on average for the three extraction methods after the MeOH washing step. However, MeOH washing was not effective to significantly reduce the amount of FFA contaminants in SPL, Ep and SUPELCO tubes.

It is noteworthy that only two out of the 24 combinations of the four sample tubes and three extraction methods showed statistically significant differences in the amount of PA or SA contaminants extracted after the MeOH washing step. The average standard deviations of exogenous FFAs for untreated GLASSCO tubes were 3–5 times higher than those of MeOH washed GLASSCO tube. These results imply that washing GLASSCO tubes with MeOH lowers the standard deviation of the measurement of FFA contaminants, leading to the improved precision in the quantification of FFAs. However, no significant difference in the standard deviation of the measured FFA contaminants was observed for the other types of tubes. Interestingly, no apparent tendency was observed in the extraction efficiency of the PA or SA contaminants in the sample tubes among the Folch, B/D, and Matyash extraction methods. On average, the Folch method extracted the highest amount of PA and SA contaminants from SPL tubes, while the Matyash method extracted the highest amount of PA or SA contaminants from Ep tubes and SUPLECO tubes. On the other hand, the relative abundance of exogenous FFAs extracted using the three methods was quite consistent between PA and SA within the same type of tube, which indicates that the extraction efficiencies of PA and SA were similar in each specific FFA extraction method.

### Quantification of PA and SA in skimmed milk

**Figure 3:**
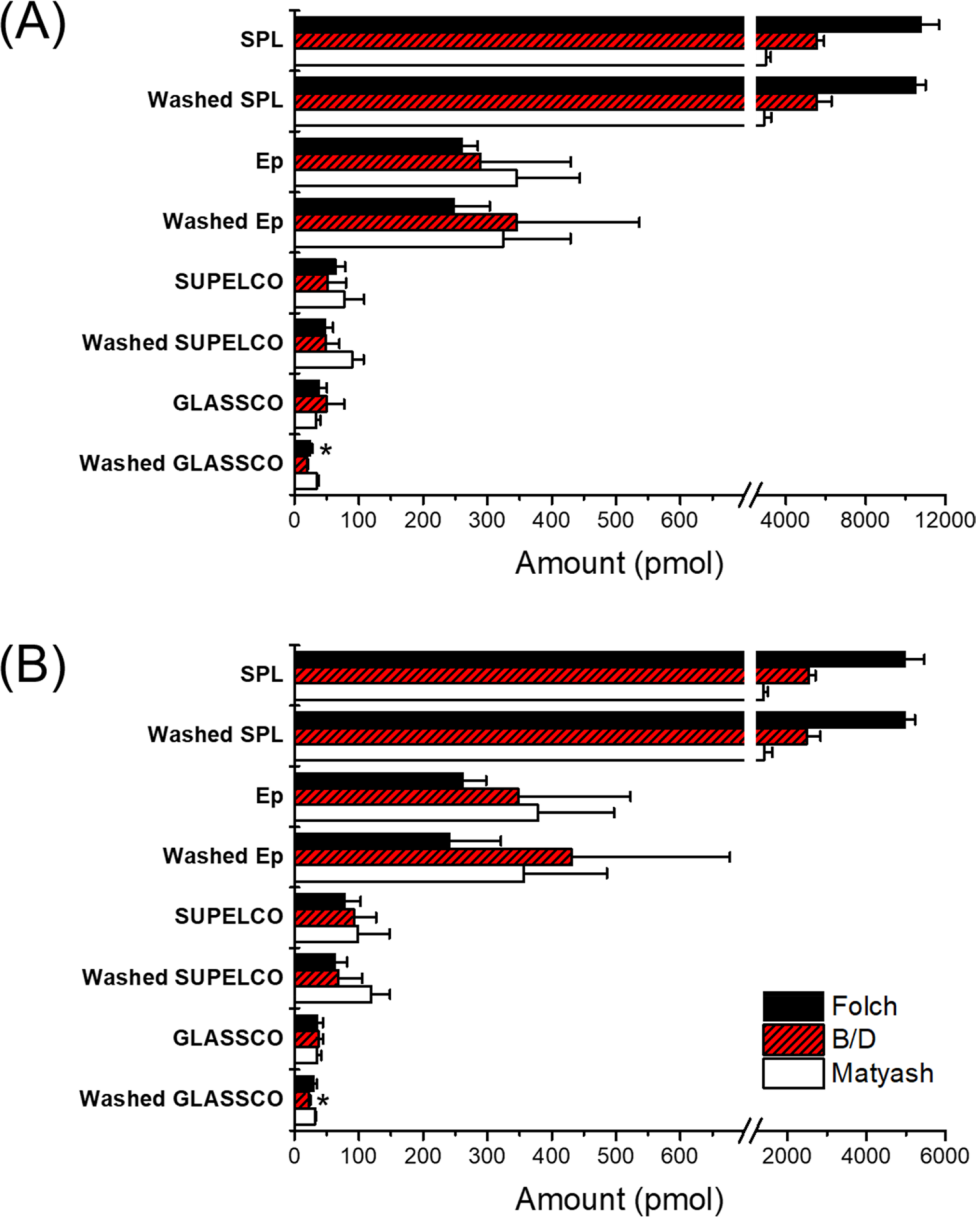
A comparison of the amount of (**A**) PA and (**B**) SA extracted from all combinations of the four sample tubes (SPL, Ep, SUPELCO and GLASCO) and three FFA extraction methods (Folch, B/D and Matyash) studied. Data are shown as the mean ± SD (*n* = 6). Asterisks denote statistically significant differences compared with the non-treatment by the Welch’s *t*-test (*P < 0.05).

The presence of exogenous FFA contaminants introduced during the sample preparation step will cause an overestimation in the quantification of FFAs in food. To assess the contribution of FFA contaminants to the analysis of the FFA content in food, two different tubes were used for FFA sampling: One was a normal Ep plastic tube with no pretreatment and the other was a GLASSCO tube rinsed three times with MeOH, which resulted in the minimum amount of residual FFAs in the experiments described in the above section. The Folch and B/D protocols were employed to extract FFAs in this study because the Matyash extraction method showed the highest PA and SA contaminants in GLASSCO tubes (Fig. 3). Skimmed milk was selected as a real food sample because of its low fat content, which can maximize the influence of exogenous contaminants on the quantification of FFAs.

The quantification results obtained for PA and SA extracted from 100 μL of skimmed milk using different combinations of the sample tubes and extraction methods are summarized in Table 2. The use of non-treated Ep plastic tubes rather than GLASSCO tubes washed with MeOH resulted in an overestimation of PA by 1.2- and 1.4-fold using the Folch and B/D processes, respectively. This also led to a 2.0- and 1.8-fold increase in SA when using the Folch and B/D methods, respectively. For comparison with the basal level of FFA contamination, the amounts of PA and SA in a blank tube without the introduction of skimmed milk were also determined. The contribution of exogenous FFA contaminants to the measured quantity of FFA from the food sample can be expressed by the ratio of the amount of FFAs in the blank tube and skimmed milk samples. The ratio of PA from an exogenous origin in the Ep tube was 45 and 31% using the Folch and B/D methods, respectively. On the other hand, it decreased to 16 and 7% using the Folch and B/D method with the MeOH washed GLASSCO tubes, respectively. In the case SA, the ratio of the residual contaminant in the plastic tube increased up to 98 and 95% using the Folch and B/D extraction methods, respectively. For the GLASSCO tube rinsed with MeOH, the residual amount of SA in skimmed milk increased by 97 and 71% using the Folch and B/D methods, respectively. In addition, the MeOH washed GLASSCO tubes showed lower standard deviations in the FFA quantification data when compared with the plastic Ep tubes, as seen above in the comparative study on the different sample extraction methods and sample tubes. These results clearly demonstrate that FFA extraction in a glass tube using MeOH washing as a pretreatment step significantly reduces the amount of exogenous contaminants, and leads to improved accuracy and precision during the quantification of the FFAs.

**Table 2:** Quantification of PA and SA in skimmed milk.

FFA	Extraction method	Ep (pmol)		MeOH washed GLASSCO (pmol)	
		Blank	Skimmed milk	Blank	Skimmed milk
PA	Folch	380 ± 130	840 ± 170	110 ± 50	680 ± 90
	B/D	310 ± 10	1000 ± 130	50 ± 10	71 ± 60
SA	Folch	650 ± 150	660 ± 120	320 ± 60	330 ± 80
	B/D	510 ± 80	530 ± 70	210 ± 40	300 ± 40

Blank indicates the amount of FFA contaminants extracted from an empty tube without the introduction of skimmed milk. Data are presented as the mean ± SD (*n* = 3).

The present study was designed to search for an optimized experimental procedure that minimizes the amount of foreign FFA contaminants negatively affecting the accurate quantification of FFAs in food. The quantities of exogenous FFAs detected using LC–MS were compared in terms of the pretreatment of the sample tube, FFA extraction method, and type of sample tube used. The combined use of MeOH washing and glassware proved to be the most effective way to reduce the amount of FFA contaminants. In particular, MeOH washing is helpful for further lowering the amount of PA and SA contaminants in glass tubes where the amount of exogenous FFAs is typically at the sub-nanomolar level before MeOH washing. It is strongly recommended to avoid the use of plastic consumables that are expected to be the main source of exogenous FFA contaminants during the analysis of FFAs in food.

## Supplementary Information

Supplementary Information
