# Supplementary material for: Development of an optimized sample preparation method for quantification of free fatty acids in food using liquid chromatography-mass spectrometry
Source: Sci Rep. 2021 Mar 15;11:5947. doi: 10.1038/s41598-021-85288-1 (PMC7961064; doi:10.1038/s41598-021-85288-1)
Supplement: Supplementary file 1 — Supplementary Information [file 41598_2021_85288_MOESM1_ESM.docx]

Supporting Information

**Development of an optimized sample preparation method for quantification of fatty acids in food using liquid chromatography-mass spectrometry**

Hyejin Park^§^, Woo-Young Song^§^, Hyeonjeon Cha and Tae-Young Kim*

School of Earth Sciences and Environmental Engineering, Gwangju Institute of Science and Technology, Gwangju 61005, Republic of Korea

*Corresponding author: kimtaeyoung@gist.ac.kr

^§^These authors contributed equally: Hyejin Park and Woo-Young Song

**Table of Contents**

Figure S1. Inter-lot variation of exogenous (A) PA and (B) SA extracted from GLASSCO glass tubes with different tube pretreatment.

Table S1. Experimental conditions for comparison of different tube pretreatment methods

Table S2. Experimental conditions for comparison of three extraction methods and four types of sample tubes


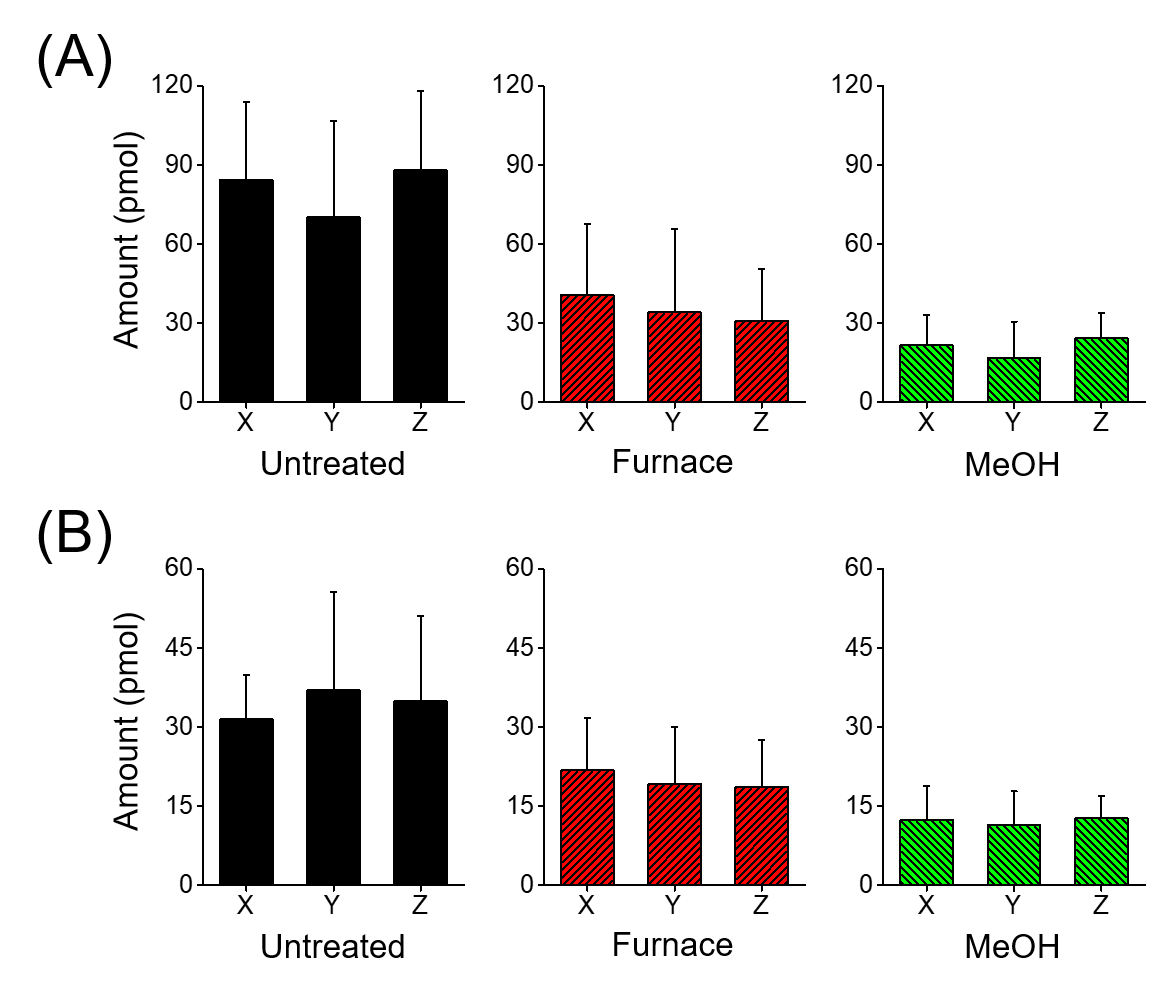


**Figure S1.** Inter-lot variation of exogenous (A) PA and (B) SA extracted from GLASSCO glass tubes with different tube pretreatment. X, Y and Z indicates three different lots, and data are shown as the mean ± deviation (*n* = 6)..

Table S1. Experimental conditions for comparison of different tube pretreatment methods

| **Conditions** | **Tube type** | **Pretreatment** | **Additive** | **Number of packages** | **Number of tubes** |
| --- | --- | --- | --- | --- | --- |
| 1 | GLASSCO | Untreated |  | 3 | 18 |
| 2 |  | Furnace |  | 3 | 18 |
| 3 |  | MeOH |  | 3 | 18 |
| 4 |  | EtOH |  | 1 | 6 |
| 5 |  | *n*-hexane |  | 1 | 6 |
| 6 |  | EtOH + *n*-hexane |  | 1 | 6 |
| 7 |  | EtOH+acid | 0.1% FA | 1 | 6 |
| 8 |  | EtOH+base | 0.1% NH4OH | 1 | 6 |

Table S2. Experimental conditions for comparison of three extraction methods and four types of sample tubes

| **Conditions** | **Tube type** | **Pretreatment** | **Extraction method** | **Number of tubes** |
| --- | --- | --- | --- | --- |
| 1 | SUPELCO | Untreated | Folch,  B/D,  Matyash | 18 |
| 2 | SUPELCO | MeOH |  | 18 |
| 3 | GLASSCO | Untreated |  | 18 |
| 4 | GLASSCO | MeOH |  | 18 |
| 5 | Ep | Untreated |  | 18 |
| 6 | Ep | MeOH |  | 18 |
| 7 | SPL | Untreated |  | 18 |
| 8 | SPL | MeOH |  | 18 |
